# Sequential Development of Iliopsoas Hematoma and Abscess in an Elderly Patient With COVID-19: A Case Report

**DOI:** 10.7759/cureus.92555

**Published:** 2025-09-17

**Authors:** Kazusa Hirokane, Natsumi Yamamoto, Shiho Amano, Kurumi Kasai, Ryuichi Ohta

**Affiliations:** 1 Community Care, Unnan City Hospital, Unnan, JPN

**Keywords:** 80 and over, aged, covid-19, family medicine, general medicine, hematoma/complications, iliopsoas abscess, iliopsoas muscles/pathology, immunosuppression, rural

## Abstract

Iliopsoas hematoma and iliopsoas abscess are rare but serious conditions that may complicate the course of elderly patients with COVID-19. The infection itself, along with corticosteroids, anticoagulation, and other immunosuppressive therapies used in treatment, can increase susceptibility to both bleeding and secondary infections. Distinguishing between hematoma and abscess is particularly challenging in this setting, as their clinical presentations often overlap. Early recognition through repeated imaging and timely intervention is therefore essential. We report the case of an 82-year-old man with COVID-19 pneumonia initially treated with dexamethasone, remdesivir, and antibiotics. His respiratory status worsened, prompting transfer to our hospital, where additional immunosuppressive therapy and anticoagulation were administered. During hospitalization, he developed acute right lower abdominal pain, and imaging revealed an iliopsoas hematoma, for which conservative management was chosen. He was later re-admitted with recurrent respiratory failure and diagnosed with organizing pneumonia requiring steroids. Subsequently, his oral intake declined, and further evaluation demonstrated a retroperitoneal mass with intralesional gas. Aspiration confirmed purulent fluid, establishing the diagnosis of an iliopsoas abscess. Drainage and antimicrobial therapy were initiated, resulting in gradual clinical improvement. This case underscores the diagnostic challenge of differentiating hematoma from abscess in elderly patients after COVID-19 and highlights the importance of vigilant follow-up.

## Introduction

Coronavirus disease 2019 (COVID-19) has diverse effects in both the acute and chronic phases [1]. During the acute stage, it causes pulmonary involvement and systemic inflammation, which can progress to multiple organ failure [2]. In later stages, COVID-19 has been associated with autoimmune disease, bone marrow suppression leading to immunosuppression, and chronic neuroinflammation contributing to psychiatric disorders [3]. These wide-ranging effects necessitate ongoing monitoring and timely intervention across the disease course.

Elderly patients are particularly vulnerable to these complications. Frailty, comorbidities, and immune decline predispose older adults to a broad spectrum of manifestations that evolve, increasing the risk of delayed diagnosis and mortality [4,5]. Careful follow-up is crucial for detecting subtle symptoms that may indicate serious conditions.

Among these, an iliopsoas hematoma is a rare but potentially life-threatening complication. It is frequently linked to anticoagulation, coagulopathy, or tissue fragility, all of which are relevant in severe COVID-19, where anticoagulants are widely administered to reduce thrombotic risk [6,7]. Nonspecific symptoms, including abdominal pain, anemia, or lower limb weakness, may delay recognition and lead to worsening outcomes.

In addition, immunosuppressive treatments used in severe COVID-19, such as corticosteroids and immunomodulators, substantially increase the risk of opportunistic infections. Secondary bacterial or fungal infections, including iliopsoas abscess, have been reported in COVID-19 patients treated with prolonged or combined immunosuppressive regimens [7,8]. The abscess often presents with vague features such as fever, malaise, or hip pain, which may overlap with other COVID-19-related symptoms, further complicating timely diagnosis [9].

Here, we report the case of an elderly male with severe COVID-19 who developed an iliopsoas hematoma requiring intensive care, followed by abscess formation necessitating surgical management. This sequential course illustrates how COVID-19 and its treatment can create conditions that predispose to both bleeding and infectious complications, emphasizing the importance of vigilant follow-up and timely recognition of nonspecific symptoms in elderly patients.

## Case presentation

An 82-year-old man developed coronavirus disease 2019 (COVID-19) pneumonia and initially presented to a local clinic. His past medical history included type 2 diabetes mellitus, chronic renal failure, hypertension, hyperuricemia, and ventricular tachycardia. His regular medications were olmesartan, esomeprazole, amlodipine, lubiprostone, mexiletine, prednisolone, tamsulosin, and trimethoprim/sulfamethoxazole. Treatment with dexamethasone (6 mg/day), remdesivir (200 mg on day 1 followed by 100 mg/day), and ampicillin/sulbactam (3 g/day) was initiated. On the fifth day of treatment, his oxygen demand increased, and chest imaging revealed worsening findings, prompting transfer to our hospital.

**Figure 1:**
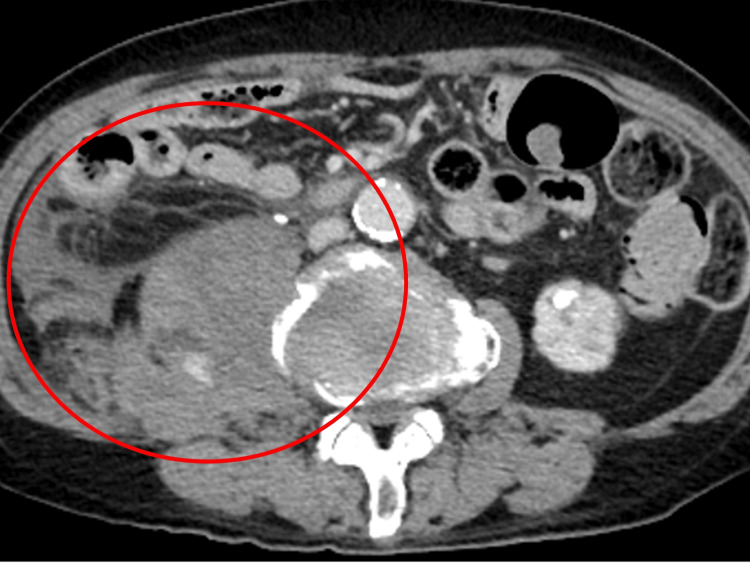
Contrast-enhanced computed tomography revealing bleeding within the right iliopsoas muscle with suspected continuous bleeding (red circle) Contrast-enhanced computed tomography demonstrated a heterogeneous hyperdense hematoma within the right iliopsoas muscle, measuring approximately 66 × 58 mm. Areas of active contrast extravasation were visible, indicating ongoing bleeding. The hematoma showed irregular margins without rim enhancement, consistent with acute intramuscular hemorrhage.

At our hospital, therapy was escalated with dexamethasone, remdesivir, tocilizumab, and baricitinib, and heparin (5,000 units subcutaneously every 12 hours) was administered for anticoagulation. During the clinical course, he developed sudden right lower abdominal pain with swelling and tenderness in the same region. Contrast-enhanced computed tomography (CT) revealed bleeding within the right iliopsoas muscle with suspected continuous bleeding, and the patient was transferred to a higher-level medical facility (Figure 1).

At the referral hospital, conservative management was chosen. Anticoagulation with heparin was immediately discontinued. The patient received transfusion support with red blood cell concentrates to correct anemia and was closely monitored in a high-care setting with serial hemoglobin measurements and repeated imaging to assess hematoma progression. Analgesics were administered for pain control, and vital signs were closely observed. Interventional radiology and surgical options were considered but deferred because of the patient’s advanced age, hemodynamic stability, and high procedural risk. However, his oxygenation subsequently worsened, and chest CT showed recurrent diffuse ground-glass opacities. He was diagnosed with organizing pneumonia following COVID-19, and steroid therapy was resumed. He was later re-transferred to our hospital for rehabilitation.

Following re-transfer, given the need for long-term disease control and to minimize corticosteroid-related adverse effects, azathioprine (25 mg/day) was initiated in combination with low-dose prednisolone (5 mg/day) as a steroid-sparing agent. However, his oral intake gradually decreased, and further evaluation was undertaken.

On admission, vital signs were as follows: blood pressure, 117/60 mmHg; pulse, 77 beats/min; temperature, 36.7 °C; and SpO₂, 93% (on room air). Cardiac examination revealed a regular rhythm without murmurs, and lung auscultation was unremarkable. Abdominal examination demonstrated right flank swelling without tenderness. There was no costovertebral angle tenderness, and no leg edema was observed.

Laboratory tests revealed elevated platelet count and C-reactive protein (CRP) (Table 1).

**Table 1:** Initial laboratory data of the patient Na, sodium; K, potassium; Cl, chloride; CRP, C-reactive protein

Parameter	Level	Reference
White blood cells	9.00	3.5–9.1 × 10^3^/μL
Neutrophils	80.9	44.0–72.0%
Lymphocytes	9.9	18.0–59.0%
Hemoglobin	10.2	11.3–15.2 g/dL
Hematocrit	30.1	33.4–44.9%
Mean corpuscular volume	93.8	79.0–100.0 fl
Platelets	37.7	13.0–36.9 × 10^4^/μL
Total protein	5.7	6.5–8.3 g/dL
Albumin	2.8	3.8–5.3 g/dL
Total bilirubin	0.5	0.2–1.2 mg/dL
Aspartate aminotransferase	9	8–38 IU/L
Alanine aminotransferase	5	4–43 IU/L
Lactate dehydrogenase	1.3	121–245 U/L
Blood urea nitrogen	23.7	8–20 mg/dL
Creatinine	1.37	0.40–1.10 mg/dL
Serum Na	135	135–150 mEq/L
Serum K	4.9	3.5–5.3 mEq/L
Serum Cl	99	98–110 mEq/L
CRP	2.77	<0.30 mg/dL

**Figure 2:**
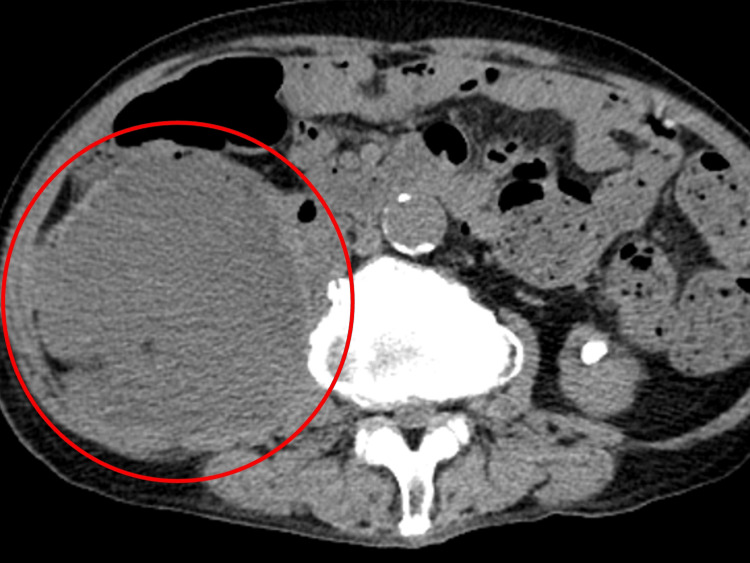
Abdominal CT revealing a well-defined, heterogeneous right retroperitoneal mass containing intralesional gas (red circle).

A non-contrast abdominal CT scan showed a well-defined, heterogeneous right retroperitoneal mass, measuring up to 85 mm in diameter, with increased density of the mass containing intralesional granular fat surrounding the fat tissue (Figure 2).

**Figure 3:**
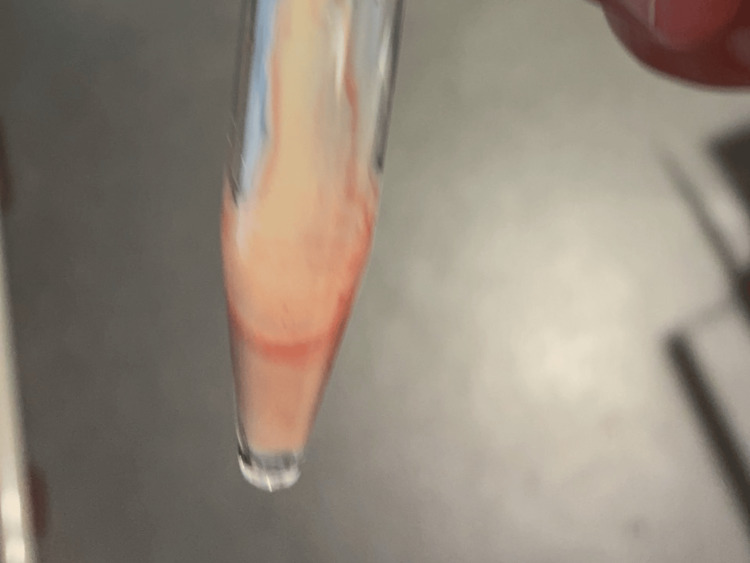
Appearance of aspirated pus.

Ultrasonography confirmed a heterogeneous mass, and percutaneous aspiration yielded purulent, foul-smelling fluid (Figure 3).

**Figure 4:**
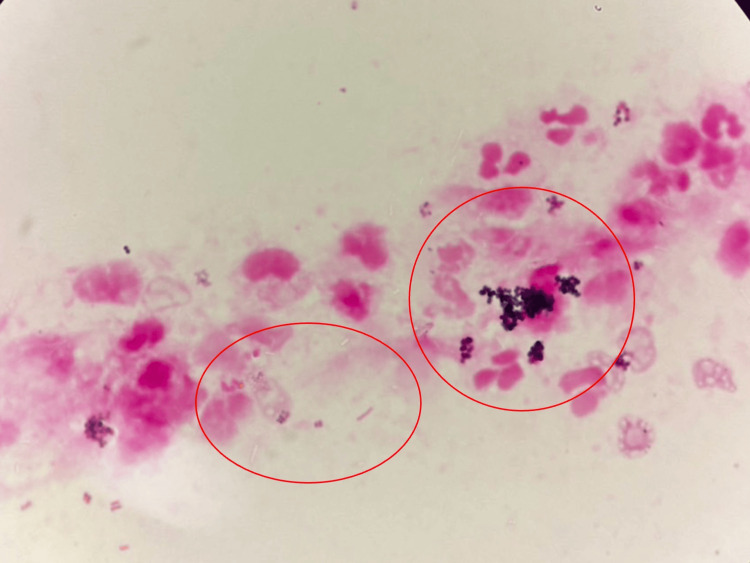
The Gram stain of the pus showing white blood cells, Gram-positive cocci, and Gram-negative rods (red circles).

The Gram stain of the pus showed white blood cells, Gram-positive cocci, and Gram-negative rods (Figure 4).

Drainage of the right iliopsoas abscess was performed, and piperacillin/tazobactam was started empirically. Cultures later identified *Staphylococcus aureus* and *Klebsiella oxytoca*, prompting a switch to trimethoprim/sulfamethoxazole, which continued for four weeks. The drain was removed on day 8 as the fluid became serous. His appetite and oral intake improved, and he was transferred to the rehabilitation ward in preparation for discharge.

## Discussion

Iliopsoas abscess is a relatively rare infectious disease, with an estimated incidence of 0.4 per 100,000 population. It often presents with nonspecific symptoms such as fever, abdominal pain, back pain, and limping, which may lead to delayed diagnosis [9]. This delay can contribute to disease progression and higher mortality, especially in elderly patients [10]. By contrast, iliopsoas hematoma typically arises in the context of anticoagulant therapy, advanced age, or coagulation disorders and is generally managed conservatively, as most cases resolve through spontaneous absorption [11]. Nevertheless, secondary infection of hematomas has been reported, resulting in abscess formation and necessitating invasive treatment [6].

Differentiating hematomas from abscesses is a clinically challenging task. On CT, both may present as heterogeneous retroperitoneal masses. Features more suggestive of abscess include intralesional gas, increased attenuation of surrounding fat, and persistent systemic inflammatory responses [12,13]. However, these findings may not always be present in the early stages. Thus, repeated imaging and invasive procedures such as aspiration and culture are crucial in patients whose clinical course does not improve with conservative management.

In the present case, initial conservative management was chosen after the diagnosis of an iliopsoas hematoma. However, upon readmission, follow-up imaging revealed intralesional gas, and aspiration confirmed purulent fluid, establishing the diagnosis of iliopsoas abscess. Initiation of targeted antimicrobial therapy combined with drainage resulted in clinical improvement. This case underscores the diagnostic difficulty in distinguishing hematoma from abscess and emphasizes the importance of timely reassessment and invasive diagnostic procedures when the clinical course is atypical.

Secondary infections following COVID-19 have been increasingly reported. Cases of bacterial and fungal abscesses, including iliopsoas abscess, have been described in patients with COVID-19 [14,15]. Importantly, in our case, COVID-19 itself was not the direct cause of the hematoma or abscess. Rather, COVID-19 created the clinical setting in which anticoagulation was administered to prevent thrombotic complications and immunosuppressive therapy was required to manage inflammation. These treatment-related factors, together with the patient’s age and comorbidities, predisposed him to both bleeding and secondary infection [16]. Elderly patients, particularly those with comorbidities, are at even greater risk due to immune senescence and chronic organ dysfunction [17]. Thus, the emphasis on COVID-19 in this report is not to suggest a direct causal role, but to highlight that the disease necessitated therapies (anticoagulation and prolonged corticosteroid/immunosuppressive treatment) that indirectly led to hematoma and abscess formation.

This case illustrates the need for heightened vigilance in elderly COVID-19 patients receiving immunosuppressive therapy. Early imaging evaluation, repeated reassessment, and timely drainage are essential for improving outcomes in patients with suspected iliopsoas abscess. General physicians dealing with various symptoms among older patients should maintain a high index of suspicion for serious infections when nonspecific symptoms arise, as early diagnosis and appropriate intervention remain critical to optimizing prognosis [18-20].

## Conclusions

Iliopsoas abscesses often present with nonspecific clinical manifestations, which can lead to delayed diagnosis. While iliopsoas hematomas generally resolve with conservative management, elderly patients and those receiving anticoagulation or immunosuppressive therapy are at increased risk of secondary infection leading to abscess formation. In such cases, careful differentiation between hematoma and abscess is essential, and repeated imaging or percutaneous aspiration should be actively employed when clinical suspicion remains. Once an abscess is confirmed, timely initiation of appropriate antimicrobial therapy and drainage can substantially improve outcomes. This case highlights the importance of maintaining vigilance for severe infections, including iliopsoas abscess, even in patients with nonspecific or vague symptoms. Particular attention should be given to those with a history of COVID-19 or immunosuppression, in whom early recognition and intervention are critical for optimizing prognosis. In such patients, COVID-19 should be understood as the background condition that necessitates high-risk therapies, rather than the direct cause of these complications.
